# New Curculionoidea (Coleoptera) records for Canada

**DOI:** 10.3897/zookeys.309.4667

**Published:** 2013-06-13

**Authors:** Hume Douglas, Patrice Bouchard, Robert S. Anderson, Pierre de Tonnancour, Robert Vigneault, Reginald P. Webster

**Affiliations:** 1 Entomology, Ottawa Plant Laboratories, Canadian Food Inspection Agency, Building 18, 960 Carling Avenue, Ottawa, ON, Canada, K1A 0C6; 2 Canadian National Collection of Insects, Arachnids and Nematodes, Agriculture and Agri-Food Canada, 960 Carling Avenue, Ottawa, Ontario, Canada, K1A 0C6; 3 Canadian Museum of Nature, P.O. Box 3443, Station D, Ottawa, Ontario, Canada, K1P 6P4; 4 22, 5e avenue, Terrasse-Vaudreuil, Quebec, Canada, J7V 3P5; 5 16 Mont St-Pierre, Oka, Quebec, Canada, J0N 1E0; 6 24 Mill Stream Drive, Charters Settlement, New Brunswick, Canada, E3C 1X1

**Keywords:** Anthribidae, Brachyceridae, Brentidae, Curculionidae, Dyophthoridae, weevils, bark beetles, pests

## Abstract

The following species of Curculionoidea are recorded from Canada for the first time, in ten cases also representing new records at the generic level: *Ischnopterapion (Ischnopterapion) loti* (Kirby, 1808); *Stenopterapion meliloti* (Kirby, 1808) (both Brentidae); *Atrichonotus taeniatulus* (Berg, 1881); *Barinus cribricollis* (LeConte, 1876); *Caulophilus dubius* (Horn, 1873); *Cionus scrophulariae* (Linnaeus, 1758); *Cryptorhynchus tristis* LeConte, 1876; *Cylindrocopturus furnissi* Buchanan, 1940; *Cylindrocopturus quercus* (Say, 1832); *Desmoglyptus crenatus* (LeConte, 1876); *Pnigodes setosus* LeConte, 1876; *Pseudopentarthrum parvicollis* (Casey, 1892); *Sibariops confinis* (LeConte, 1876); *Sibariops confusus* (Boheman, 1836); *Smicronyx griseus* LeConte, 1876; *Smicronyx lineolatus* Casey, 1892; *Euwallacea validus* (Eichhoff, 1875); *Hylocurus rudis* (LeConte, 1876); *Lymantor alaskanus* Wood, 1978; *Phloeotribus scabricollis* (Hopkins, 1916); *Scolytus oregoni* Blackman, 1934; *Xyleborus celsus* Eichhoff, 1868; *Xyleborus ferrugineus* (Fabricius, 1801); *Xylosandrus crassiusculus* (Motschulsky, 1866) (all Curculionidae). In addition the following species were recorded for the first time from these provinces and territories: Yukon – *Dendroctonus simplex* LeConte, 1868; *Phloetribus piceae* Swaine, 1911 (both Curculionidae); Northwest Territories – *Loborhynchapion cyanitinctum* (Fall, 1927) (Brentidae); Nunavut – *Dendroctonus simplex* LeConte, 1868 (Curculionidae); Alberta – *Anthonomus tectus* LeConte, 1876; *Promecotarsus densus* Casey, 1892; *Dendroctonus ponderosae* Hopkins, 1902; *Hylastes macer* LeConte, 1868; *Rhyncolus knowltoni* (Thatcher, 1940); *Scolytus schevyrewi* Semenov Tjan-Shansky, 1902 (all Curculionidae); Saskatchewan – *Phloeotribus liminaris* (Harris, 1852); *Rhyncolus knowltoni* (Thatcher, 1940); *Scolytus schevyrewi* Semenov Tjan-Shansky, 1902 (all Curculionidae); Manitoba – *Cosmobaris scolopacea* Germar, 1819; *Listronotus maculicollis* (Kirby, 1837); *Listronotus punctiger* LeConte, 1876; *Scolytus schevyrewi* Semenov Tjan-Shansky, 1902; *Tyloderma foveolatum* (Say, 1832); (all Curculionidae); Ontario – *Trichapion nigrum* (Herbst, 1797); *Nanophyes marmoratus marmoratus* (Goeze, 1777) (both Brentidae); *Asperosoma echinatum* (Fall, 1917); *Micracis suturalis* LeConte, 1868; *Orchestes alni* (Linnaeus, 1758); *Phloeosinus pini* Swaine, 1915; *Scolytus schevyrewi* Semenov Tjan-Shansky, 1902; *Xyleborinus attenuatus* (Blandford, 1894) (all Curculionidae); Quebec – *Trigonorhinus alternatus* (Say, 1826); *Trigonorhinus tomentosus tomentosus* (Say, 1826) (both Anthribidae); *Trichapion nigrum* (Herbst, 1797); *Trichapion porcatum* (Boheman, 1839); *Nanophyes marmoratus marmoratus* (Goeze, 1777) (all Brentidae); *Lissorhoptrus oryzophilus* Kuschel, 1952 (Brachyceridae); *Acalles carinatus* LeConte, 1876; *Ampeloglypter ampelopsis* (Riley, 1869); *Anthonomus rufipes* LeConte, 1876; *Anthonomus suturalis* LeConte, 1824; *Ceutorhynchus hamiltoni* Dietz, 1896; *Curculio pardalis* (Chittenden, 1908); *Cyrtepistomus castaneus* (Roelofs, 1873); *Larinus planus* (Fabricius, 1792); *Mecinus janthinus* (Germar, 1821); *Microhyus setiger* LeConte, 1876; *Microplontus campestris* (Gyllenhal, 1837); *Orchestes alni* (Linnaeus, 1758); *Otiorhynchus ligustici* (Linnaeus, 1758); *Rhinusa neta* (Germar, 1821); *Trichobaris trinotata* (Say, 1832); *Tychius liljebladi* Blatchley, 1916; *Xyleborinus attenuatus* (Blandford, 1894); *Xyleborus affinis* Eichhoff, 1868 (all Curculionidae); *Sphenophorus incongruus* Chittenden, 1905 (Dryophthoridae); New Brunswick – *Euparius paganus* Gyllenhal, 1833; *Allandrus populi* Pierce, 1930; *Gonotropis dorsalis* (Thunberg, 1796); *Euxenus punctatus* LeConte, 1876 (all Anthribidae); *Loborhynchapion cyanitinctum* (Fall, 1927) (Brentidae); *Pseudanthonomus seriesetosus* Dietz, 1891; *Curculio sulcatulus* (Casey, 1897); *Lignyodes bischoffi* (Blatchley, 1916); *Lignyodes horridulus* (Casey, 1892); *Dietzella zimmermanni* (Gyllenhal, 1837); *Parenthis vestitus* Dietz, 1896; *Pelenomus squamosus* LeConte, 1876; *Psomus armatus* Dietz, 1891; *Rhyncolus macrops* Buchanan, 1946; *Magdalis inconspicua* Horn, 1873; *Magdalis salicis* Horn, 1873 (all Curculionidae); Nova Scotia – *Dryocoetes autographus* (Ratzeburg, 1837); *Ips perroti* Swaine, 1915; *Xyleborinus attenuatus* (Blandford, 1894) (all Curculionidae); Prince Edward Island – *Dryocoetes caryi* Hopkins, 1915 (Curculionidae); Newfoundland – *Scolytus piceae* (Swaine, 1910) (Curculionidae).

Published records of *Dendroctonus simplex* LeConte, 1868 from Northwest Territories should be reassigned to Nunavut, leaving no documented record for NWT. Collection data are provided for eight provincial and national records published without further information previously.

## Introduction

Routine weevil and bark beetle identifications from plant health surveys, amateur collectors, public inquiries, and museum survey specimens regularly produce new faunal records for Canada, its provinces, and territories. The most recent checklist of the Canadian fauna is McNamara (1991).

The present article presents new findings with associated collection data so that the records may be documented with verifiable voucher specimens. These records are also reflected in the updated checklist of Canadian beetles (Bousquet et al. 2013).The following list of 97 new records are organised according to the family–group classification of Bouchard et al. (2011). We record two Brentidae, and 22 Curculionidae species new to Canada, and 72 new provincial and territorial records; many of these are of beneficial or economic pest species.

## Materials and methods

Specimens were identified (or identifications confirmed) by recognized specialists in those taxa. These are as follows: Curculionidae (Scolytinae) (Hume Douglas, Donald E. Bright); Curculionidae (other than Scolytinae), Anthribidae, Brachyceridae (Robert S. Anderson, Patrice Bouchard); Brentidae (Apioninae) and Curculionidae (Baridinae) (Jens Prena). Specimens are deposited in collections listed with the specimen data for each species.

All collections listed below were reviewed by one or more authors for undocumented curculionoid records except for DEBU, GLFC, and City of Saskatoon. For these three collections we included only the specimens identified as new by their staff. It is possible that additional undocumented curculionoid records remain in most of the collections listed below.

The use of term adventive used here follows that of Wheeler and Hoebeke (2009). Such adventive species are non-natives with established North American populations, intentionally or accidentally introduced by humans, effectively since the first arrival of Europeans.

### Canadian collections that provided material cited here:

**AFC** Atlantic Forestry Centre, Natural Resources Canada, Canadian Forest Service, Fredericton, New Brunswick

**CCCH** Claude Chantal Insect Collection (personal collection), Varennes, Quebec

**City of Saskatoon** Saskatchewan (Contact Jeff Boone)

**CHMS** Henri Miquet-Sage Insect Collection (personal collection), Mont-Saint-Hilaire, Quebec

**CMNC** Canadian Museum of Nature, Ottawa, Ontario

**CNCI** Canadian National Collection of Insects, Arachnids, and Nematodes, Agriculture and Agri-Food Canada Research Centre, Ottawa, Ontario

**CPTO** Pierre de Tonnancour Insect Collection (personal collection), Terrasse-Vaudreuil, Quebec

**CRLI** René Limoges Insect Collection (personal collection), Montreal, Quebec

**CRVI** Robert Vigneault Insect Collection (personal collection), Oka, Quebec

**CSLA** Serge Laplante Insect Collection (personal collection), Gatineau, Quebec

**DEBU** University of Guelph Insect Collection, Guelph, Ontario

**GLFC** Great Lakes Forestry Centre, Sault Ste. Marie, Ontario

**LEMQ** Lyman Entomological Museum, McGill University, Ste-Anne-de-Bellevue, Quebec

**NBM** New Brunswick Museum, Saint John, New Brunswick

**RWC** Reginald P. Webster Collection (personal collection), Charters Settlement, New Brunswick

## Results

### 1) Family Anthribidae Billberg, 1820 Subfamily Anthribinae Billberg, 1820 Tribe Cratoparini LeConte, 1876

#### Euparius paganus

Gyllenhal, 1833 new to New Brunswick, and new data supporting first records for Canada

##### Note.

This native fungus weevil was recorded “from Quebec to Florida, west to Iowa, Kansas and Texas” by Valentine (1999) without specific details about its distribution within Quebec. We provide those data here for the first time and provide data for specimens from New Brunswick.

##### Specimen data.

**New Brunswick:** Carleton County, Jackson Falls, Bell Forest, 46.2200°N, 67.7231°W, 17–31.vii.2012, 31.vii-14.viii.2012, C. Alderson & V. Webster, Lindgren traps in canopy of *Juglans cinerea* and *Tilia americana* (1, AFC; 5, RWC). **Quebec:** MRC Vaudreuil-Soulanges, Rigaud, 12.vii, 14.vii.1998 (2, CRVI); MRC Deux-Montagnes, Oka, Mont St-Pierre, 19.v., 2.viii.2003, UV Light, R. Vigneault (2, CRVI); MRC Les Collines-de-l’Outaouais, Eardley, “petite colline d’Eardley”, 17.vi., 25.vii.2003, 14.v.2004, S. Laplante, R. Vigneault, at UV light (3, CSLA; 2 CRVI); MRC Les Collines-de-l’Outaouais, Eardley, “petite colline d’Eardley”, 25.vii.2003, S. Laplante, on dead branch of *Prunus pensylvanica* at night (1, CSLA); MRC Vaudreuil-Soulanges, Terrasse-Vaudreuil, 3.vii.2011 (01:00), white and UV lights, P. de Tonnancour (1, CPTO); MRC Vaudreuil-Soulanges, Terrasse-Vaudreuil, 5.vii.2012 (23:00), white and UV lights, P. de Tonnancour (1, CPTO); MRC Vaudreuil-Soulanges, Terrasse-Vaudreuil, 27.vii.2012, at night (22:30), white and mercury lights, P. de Tonnancour (1, CPTO).

### Tribe Stenocerini Kolbe, 1895

#### Allandrus populi

Pierce, 1930 new to New Brunswick

##### Note.

This transcontinental Canadian species appears to be associated with *Populus tremuloides* (Bright 1993).

##### Specimen data.

**New Brunswick:** Carleton County, Meduxnekeag Valley Nature Preserve, 46.1907°N, 67.6740°W, 3–17.vii.2012, C. Alderson & V. Webster, Lindgren trap in *Populus tremuloides* canopy (3, AFC; 3, RWC); Sunbury County, Gilbert Island, 45.8770°N, 66.2954°W, 11–29.vi–11.2012, 11–25.vii.2012, 25.vii–8.viii.2012, 8–21.viii.2012 Lindgren traps in *Populus tremuloides* canopy, C. Alderson, C. Hughes & V. Webster (4, AFC; 1, NBM; 5, RWC); York County, 15 km W of Tracy, off Rt. 645, 45.6848°N, 66.8821°W, 16–30.vi.2010, Lindgren funnel trap, R. Webster & C. MacKay (1, RWC).

### Tribe Trigonorhinini Valentine, 1999

#### Trigonorhinus alternatus

(Say, 1826) new to Quebec

##### Note.

This fungus weevil was recorded in Canada from Alberta, Manitoba and Ontario by McNamara (1991).

##### Specimen data.

**Quebec:** MRC Marguerite-D’Youville, Varennes (Verchères), 6.vi.2003, C. Chantal (1, CCCH).

#### Trigonorhinus tomentosus tomentosus

(Say, 1826) new to Quebec

##### Note.

This native species was only recorded in Canada from Ontario by McNamara (1991).

##### Specimen data.

**Quebec:** Montreal, 23.vii.1967, E. J. Kiteley (1, CNCI); Montreal, Sainte- Anne-de-Bellevue, 5.ix.1967, W. Boyle (1, LEMQ; 1, CMNC); Montreal, 21.viii.1968 (1, CNCI); Montreal, 26.viii.1968 (2, CNCI); MRC Vaudreuil-Soulanges, Rigaud (4 mi. S.E.), 4.vii.1972, C. Boyle (1, LEMQ); RCM Le Haut-Saint-Laurent, Cairnside, 29.viii.1981 (1, CNCI); MRC Vaudreuil-Soulanges, Notre-Dame-de-l’Île-Perrot, 27.v.2011 (13:00), 10.vi.2011 (17:00), 2.vii.2011 (15:00), swept from grasses, swept from *Scirpus* sp., ex. flowers of *Lythrum salicaria*, P. de Tonnancour (5, CPTO); Montreal, Sainte-Anne-de-Bellevue, 24.viii.2011 (13:00), swept from *Ambrosia artemisiifolia*, P. de Tonnancour (3, CPTO); Montreal, Sainte-Anne-de-Bellevue, 12.ix.2011 (12:00), swept from *Ambrosia artemisiifolia*, P. de Tonnancour (1, CPTO); Montreal, Sainte-Anne-de-Bellevue, 5.vii.2012 (13:00), swept from forbs, P. de Tonnancour (1, CPTO).

### Tribe Tropiderini Lacordaire, 1865

#### Gonotropis dorsalis

(Thunberg, 1796) new to New Brunswick

##### Note.

This transcontinental Canadian species has previously been placed in the genus *Tropideres* Schönherr.

##### Specimen data.

**New Brunswick:** York County, Charters Settlement, 45.8395°N, 66.7391°W, 20.v.2012, R Webster, on window screen (1, RWC). Fredericton, Odell Park, 45.9571°N, 66.6650°W, 10–26.vii.2012, C. Alderson & V. Webster, old-growth eastern hemlock forest, Lindgren trap in *Tsuga canadensis* canopy (2, RWC).

### Tribe Zygaenodini Lacordaire, 1865

#### Eusphyrus walshii

LeConte, 1876 new data supporting first records for Quebec

##### Note.

This species was recorded “from Quebec to Florida, west to Michigan and eastern Texas” by Valentine (1999, not McNamara 1991) without specific details about its distribution within Quebec. We provide data on the occurence of this species in Quebec for the first time.

##### Specimen data.

**Quebec:** MRC Les Collines-de-l’Outaouais Luskville, Chemin Pilon, 24.vi.2001, C. Chantal (1, CCCH); MRC Marguerite-D’Youville, Varennes (Verchères), 9.ix.2002, C. Chantal (1, CCCH); Longueuil, St-Bruno-de-Montarville, 45.588°N, 73.303°W, 22–29.vii.2008, Projet Défense Nationale, Site 1 Parcelle 4, érablière à caryer, Sante trap, Propylene 100%, 2008-3-1437 (1, CNCI); MRC Vaudreuil-Soulanges, Rigaud, 1.vii.1993 (15: 00), beaten from dead branch of *Carya ovata*, P. de Tonnancour (1, CPTO); Montreal, Ste-Anne-de-Bellevue, 5.vii.2012 (15:00) on *Ulmus americana*, P. de Tonnancour (1, CPTO); MRC Marguerite-D’Youville, Contrecoeur, 7.vii.2012, dead branch of *Salix* sp., P. de Tonnancour (1, CPTO).

### Subfamily Choraginae Kirby, 1819 Tribe Araecerini Lacordaire, 1865

#### Euxenus punctatus

LeConte, 1876 new to New Brunswick

##### Note.

This species was previously known in Canada only from Quebec (McNamara 1991). It is the smallest anthribid in Canada.

##### Specimen data.

**New Brunswick:** Queens County, Jemseg, 45.8412°N, 66.1195°W, 28.vi–10.vii.2012, Lindgren trap under *Quercus macrocarpa*, C. Hughes & R. Webster (1, RWC).

### 2) Family Brentidae Billberg, 1820 Subfamily Apioninae Schönherr, 1823 Tribe Apionini Schönherr, 1823

#### Loborhynchapion cyanitinctum

(Fall, 1927) new to New Brunswick, Northwest Territories

##### Note.

This widespread and northern species is recorded from the maritime provinces for the first time. It has been collected on *Astragalus* (Bright 1993).

##### Specimen data.

**New Brunswick:** Carleton County, Meduxnekeag Valley Nature Preserve, 46.1891°N, 67.6762°W, 11.vi.2012, swept from foliage by river, R.P. Webster (1, RWC). **Northwest Territories:** Anderson River Delta, Fox Den II, 29.vi–15.vii.1977, D. Shpeley & G.E. Ball (1, CMNC).

#### Ischnopterapion (Ischnopterapion) loti

(Kirby, 1808) new to Canada

##### Note.

This adventive species is broadly distributed in the Palaearctic Region (Alonso-Zarazaga 2011). In Canada it feeds on the introduced weed *Lotus corniculatus* L. (Fabaceae) which is common in eastern Canada and British Columbia (Turkington and Franko 1980). This species may be more widespread in Canada than presently documented because extensive surveys have not been conducted.

##### Specimen data.

**Quebec:** MRC Vaudreuil-Soulanges, Ville de l’Île-Perrot, 17.v.2011, swept from *Lotus corniculatus*, P. de Tonnancour (2, CNCI; 17, CPTO); MRC Vaudreuil-Soulanges, Ville de l’Île-Perrot, 18.v.2011, swept from *Hesperis matronalis*, P. de Tonnancour (5, CPTO).

#### Stenopterapion meliloti

(Kirby, 1808) new to Canada

##### Note.

This adventive species is broadly distributed in the Palaearctic Region (Alonso-Zarazaga 2011). In Canada it feeds on the introduced weed *Melilotus alba* Desr. which is common across Canada (Turkington et al. 1978). This species may be more widespread in Canada than presently documented because extensive surveys have not been conducted.

##### Specimen data.

**Quebec:** MRC La Vallée-du-Richelieu, Mont-Saint-Hilaire, 7.vi., 23.vi.2004, 18.viii.2007, H. Miquet-Sage (3, CHMS); MRC Vaudreuil-Soulanges, Notre-Dame-de-l’Île-Perrot, 2.iv.2007, 12.v.2011 (13:00), 17.v.2011, 10.vi.2011, 14.ix.2011, 19.iv.2012 (13:00), under bark of *Pinus strobus*, swept from *Melilotus alba*, P. de Tonnancour (2, CNCI; 29, CPTO).

#### Trichapion nigrum

(Herbst, 1797) new to Ontario and Quebec

##### Note.

This native species was newly recorded in Canada in New Brunswick by Majka et al. (2007a) and feeds on the seeds of *Robinia pseudoacacia* L. (Fabaceae). That the first specimen was collected over 50 years ago, that the host is widespread, and that this tribe has received little taxonomic attention, all suggest that this species may be more widespread than known.

##### Specimen data.

**Ontario:** Simcoe Co., Cookstown, Lake Simcoe, 11.vi.1962, S62-1237-01, *Robinia pseudoacacia*, F. I. S. (1, CNCI); **Quebec:** MRC Vaudreuil-Soulanges, Ville de l’Île-Perrot, 21.vi.2011 (14:00) beaten from *Robinia pseudoacacia*, P. de Tonnancour (2, CNCI; 1, CPTO); MRC Vaudreuil-Soulanges, Ville de l’Île-Perrot, 1.vii.2011 (18:00) beaten from *Robinia pseudoacacia*, P. de Tonnancour (14, CNCI).

#### Trichapion porcatum

(Boheman, 1839) new to Quebec

##### Note.

This native species was recorded from Ontario by McNamara (1991) and from New Brunswick by Webster et al. (2012).

##### Specimen data.

**Quebec:** Gatineau, Queens Park, 9.vii.2011 (19:00) beaten from *Desmodium canadense*, P. de Tonnancour (2, CNCI; 1, CPTO); MRC Vaudreuil-Soulanges, Ville de l’Île-Perrot, 21.vi.2011 (14:00) beaten from *Robinia pseudoacacia*, P. de Tonnancour (1, CPTO).

### Subfamily Nanophyinae Gistel, 1848 Tribe Nanophyini Gistel, 1848

#### Nanophyes marmoratus marmoratus

(Goeze, 1777) new to Ontario and Quebec

##### Note.

This species was introduced to New York State for the biological control of purple loosestrife, *Lythrum salicaria* L. (Lythraceae) (Anderson 2003).It is likely that the new Ontario and Quebec records represent natural dispersal from the adjacent northern USA.

##### Specimen data.

**Ontario:** Lanark Co., Packenham, 10.ix.2012, on *Lythrum salicaria*, E. St-Louis (1, CNCI); **Quebec:** MRC Vaudreuil-Soulanges, Saint-Lazare, 3.vi.2011, 1.ix.2011, swept or beaten from *Lythrum salicaria*, P. de Tonnancour (2, CPTO); MRC Vaudreuil-Soulanges, Notre-Dame-de-l’Île-Perrot, 19.v., 22.v., 30.v., 31.v.2011, 2.vi.2011, 2.vii.2011 (15:00), 29.viii.2011, 2.ix.2011, 1.vi.2012, all beaten or swept from *Lythrum salicaria*, P. de Tonnancour (18, CPTO; 23 CNCI); MRC Vaudreuil-Soulanges, Mt. Rigaud, 19.iv.2012, beaten from *Salix* sp., P. de Tonnancour (1, CPTO); MRC Les Collines-de-l’Outaouais, Bristol, Knox Landing Road, sand pit, 26.v.2012, on *Lythrum salicaria*, S. Laplante (2, CNCI).

### 3) Family Dryophthoridae Schönherr, 1825 Subfamily Rhynchophorinae Schönherr, 1833 Tribe Sphenophorini Lacordaire, 1865

#### Sphenophorus incongruus

Chittenden, 1905 new to Quebec

##### Note.

This native species was recorded from Ontario by McNamara (1991), and is associated with great rush, *Schoenoplectus tabernaemontani* (C.C.Gmel.) Palla, in wetlands (Vaurie 1951).

##### Specimen data.

**Quebec:** MRC Deux-Montagnes, Oka, Parc national d’Oka, 7.iv.1991, under stone, lakeside, P. de Tonnancour (1, CPTO); MRC Deux-Montagnes, Oka, Parc Paul-Sauvé, 9.v.1993, 13.v.1994, P. Vigneault (2, CRVI).

### 4) Family Brachyceridae Billberg, 1820 Subfamily Erirhininae Schönherr, 1825 Tribe Stenopelmini LeConte, 1876

#### Lissorhoptrus oryzophilus

Kuschel, 1952 new to Quebec

##### Note.

This native species was previously known from Alberta (McNamara 1991). A pest of cultivated rice (where grown); larvae feed externally on roots (Anderson 2002). It is likely that related native semiaquatic grasses are the hosts elsewhere in North America.

##### Specimen data.

**Quebec:** MRC Marguerite-D’Youville, Varennes (Verchères), 25.vi.2007, C. Chantal (1, CCCH).

### 5) Family Curculionidae Latreille, 1802 Subfamily Curculioninae Latreille, 1802 Tribe Anthonomini Thomson, 1859

#### Anthonomus rufipes

LeConte, 1876 new to Quebec

##### Note.

This native species was recorded from Alberta by McNamara (1991). Based on label data for specimens reported here, the species appears to be associated with *Aster* and *Symphyotrichum* spp. (Asteraceae).

##### Specimen data.

**Quebec:** RCM Rouville, Rougemont, 4.vi.1966, C. Chantal (2, CCCH); MRC La Vallée-du-Richelieu, Saint-Lambert, 8.viii.1966, 16.vii.1967, P. de Tonnancour (3, CPTO); MRC L’Islet, Lac des Trois-Saumons, 1.vii.1968, C. Chantal (1, CCCH); Montreal, Dollard-des-Ormeaux, 25.viii.1974, C. Chantal (7, CCCH); MRC Vaudreuil-Soulanges, Terrasse-Vaudreuil, 17.vi.1993 (18:00), apical bud of *Symphyotrichum novae-angliae*, P. de Tonnancour (7, CPTO); MRC La Vallée-du-Richelieu, Mont-Saint-Hilaire, 9.vi.2004, H. Miquet-Sage (1, CHMS); MRC Vaudreuil-Soulanges, Notre-Dame-de-l’Île-Perrot, 31.v.2011, 1.vi., 2.vi., 17.vi.2011 (13:00, 14:00, 15:00), swept from *Solidago/Aster*, P. de Tonnancour (12, CPTO); MRC Vaudreuil-Soulanges, Ville de l’Île-Perrot, 4 June 2011 (11:00), swept from *Symphyotrichum novae-angliae*, P. de Tonnancour (3, CPTO); MRC Vaudreuil-Soulanges, Notre-Dame-de-l’Île-Perrot, 17.vi.2011 (13:00), swept from *Aster* sp., P. de Tonnancour (1, CPTO); MRC Vaudreuil-Soulanges, Ville de l’Île-Perrot, 13 August 2011 (13:00), swept from *Aster* sp., P. de Tonnancour (2, CPTO); MRC Vaudreuil-Soulanges, Notre-Dame-de-l’Île-Perrot, 1.vi.2012 (13:00), swept from *Trifolium pratense*, P. de Tonnancour (1, CPTO); MRC Vaudreuil-Soulanges, Saint-Lazare, 31.viii.2012, swept from *Symphyotrichum novae-angliae*, P. de Tonnancour (1, CPTO).

#### Anthonomus suturalis

LeConte, 1824 new to Quebec

##### Note.

This species was recorded from British Columbia and Ontario by McNamara (1991). The species is associated with *Phyloxera* galls on leaves of *Carya* spp. (Juglandaceae) (Ahmad and Burke 1972).

##### Specimen data.

**Quebec:** MRC Vaudreuil-Soulanges, Rigaud, 17.vii.1979, S. Laplante (1, CSLA); MRC Deux-Montagnes, Oka, Deux-Montagnes, beaten from *Carya ovata*, 13.vii., 15.vii.1982, 21.v.1983, C. Chantal (9, CCCH); Montreal, Dollard-des-Ormeaux, 16.vii.1982, C. Chantal (1, CCCH); Montreal, Sainte-Anne-de-Bellevue, 12.vi.1984, M.C. Larivière (3, LEMQ); Laval, Ste. Dorothée, 10.v.1987, F. Genier (1, CMNC); MRC Vaudreuil-Soulanges, Rigaud, 16.vi.1990, on *Carya ovata*, S. Laplante (1, CSLA); MRC Vaudreuil-Soulanges, Rigaud, 8.vii.1990 (15:00), beaten from *Carya ovata*, P. de Tonnancour (5, CPTO); Montreal, Sainte-Anne-de-Bellevue, 13.v.1992, S. Côté (1, CMNC); MRC Deux-Montagnes, Oka, 14.v.1993, 10.vii.1996, R. Vigneault (6, CRVI); MRC Deux-Montagnes, Oka, Parc d’Oka, Calvaire d’Oka, 5.vi.2004, R. Vigneault (1, CRVI); MRC Vaudreuil-Soulanges, Notre-Dame-de-l’Île-Perrot, 12.v.2011 (14:00), beaten from *Rubus* sp., P. de Tonnancour (1, CPTO); MRC Vaudreuil-Soulanges, Terrasse-Vaudreuil, 19.v.2011, 21.v.2011 (14:00, 23:00), beaten from *Prunus nigra*, white and UV light, P. de Tonnancour (2, CPTO).

#### Anthonomus tectus

LeConte, 1876 new to Alberta

##### Note.

This species is known in Canada only from the prairie provinces of Manitoba and Saskatchewan; we here add Alberta and document an association with *Heterotheca villosa* (Pursh.) Shinners (Asteraceae).

##### Specimen data.

**Alberta:** 6.5 km E. Clyde, 15.vii.1989, swept from *Heterotheca villosa* (Pursh.) Shinners, R.S. Anderson (5, CMNC).

#### Pseudanthonomus seriesetosus

Dietz, 1891 new to New Brunswick

##### Note.

This eastern North American species is now recorded from New Brunswick. Adults have been associated with *Vaccinium* sp. (Ericaceae) (Clark 1987).

##### Specimen data.

**New Brunswick:** Restigouche County, Dionne Brook P.N.A., 47.9030°N, 68.3503°W, 30.v–15.vi.2011, Lindgren funnel trap, M. Roy & V. Webster (1, CMNC; 1, RWC).

### Tribe Cionini Schönherr, 1825

#### Cionus scrophulariae

(Linnaeus, 1758) new to Canada

##### Note.

This adventive Palaearctic species, which is associated with *Scrophularia* and *Verbascum* (Scrophulariaceae), is known to be established in New York (Anderson 2002).

##### Specimen data.

**Quebec:** Montreal,19.vi.2009, on *Verbascum thapsus*, CERL 15531, R. Limoges (1, CRLI).

### Tribe Curculionini Latreille, 1802

#### Curculio pardalis

(Chittenden, 1908) new to Quebec

##### Note.

This native species was recorded in Canada from Manitoba and Ontario by McNamara (1991). It is is associated with *Quercus* spp. (Fagaceae) throughout its range (Gibson 1969).

##### Specimen data.

**Quebec:** MRC Deux-Montagnes, Oka, Parc d’Oka, 6.vi.1995, R. Vigneault (1, CRVI); Parc Gatineau, Mont King, 2.vii.1995, R. Vigneault (2, CRVI); MRC Les Collines-de-l’Outaouais, Luskville, 18.vii.1996, R. Vigneault (1, CRVI); MRC Deux-Montagnes, Oka, 11.vi.1997, R. Vigneault (2, CRVI); MRC Vaudreuil-Soulanges, Rigaud, 12.vii.1997, UV Light, R. Vigneault (1, CRVI); MRC Les Collines-de-l’Outaouais, Eardley, 19.vii.1997, R. Vigneault (1, CRVI).

#### Curculio sulcatulus

(Casey, 1897) new to New Brunswick

##### Note.

This eastern North American species is associated with *Quercus* spp. throughout its range (Gibson 1969).

##### Specimen data.

**New Brunswick:** Queens County, Jemseg, 45.8412°N, 66.1195°W, 21.viii.-7.ix.2012, Lindgren trap in *Quercus rubra* canopy, C. Hughes & K. Van Rooyen (1, RWC); Sunbury County, Sunpoke Lake, 45.7656°N, 66.5550°W, 15–27.viii.2012, Lindgren trap under *Quercus rubra*, C. Alderson & V. Webster(1, RWC).

### Tribe Mecinini Gistel, 1848

#### Mecinus janthinus

(Germar, 1821) new to Quebec

##### Note.

This adventive Palaearctic stem-mining weevil was introduced for the biological control of toadflaxes, *Linaria* spp. (Scrophulariaceae), and was known from Alberta, British Columbia, and Nova Scotia (Majka et al. 2007b, De Clerck-Floate and Cárcamo 2011).

##### Specimen data.

**Quebec:** MRC Marguerite-D’Youville, Varennes (Verchères), 30.vii.2011, 9.v., 12.v., 14.v., 24.v.2012, 1.vi.2012, C. Chantal (11, CCCH); MRC Pierre-De Saurel, Sorel-Tracy, 14.v.2012, C. Chantal (1, CCCH).

#### Rhinusa neta

(Germar, 1821) new to Quebec

##### Note.

This adventive Palaearctic species was introduced into British Columbia for control of toadflaxes, *Linaria* spp., (De Clerck-Floate and Cárcamo 2011).

##### Specimen data.

**Quebec:** MRC Deux-Montagnes, Oka, Parc d’Oka, Calvaire d’Oka, 5.v.2000, R. Vigneault (1, CRVI).

### Tribe Rhamphini Rafinesque, 1815

#### Orchestes alni

(Linnaeus, 1758) new to Ontario and Quebec

##### Note.

In Canada this species was previously only known from British Columbia. Adults and larvae of this adventive species are associated with *Ulmus americana* L. (Ulmaceae) (Anderson *et al*. 2007).

##### Specimen data.

**Ontario:** Toronto, Yonge Street and York Mills Road, 30.v.2008, on *Ulmus* leaves, C. Grant (CFIA) (6, CNCI); Essex Co., Leamington, 17.v.2011, in greenhouse, Dean coll. (1, CNCI); Essex Co., Windsor, Malden Park, 17.iv.2012, Forestry Trapping, CFIA (1, CNCI); **Quebec:** MRC Le Haut-Saint-François, Scotstown, 16.vii.2007, C. Levesque (1, CNCI); Longueuil, 18.vi.2011 (18:00), swept from *Ambrosia artemisiifolia*, P. de Tonnancour (1, CPTO); Montreal, Sainte-Anne-de-Bellevue, 2.vii.2011 (13:00) beaten from *Ulmus americana*, P. de Tonnancour (10, CNCI; 14, CPTO); Montreal, Sainte-Anne-de-Bellevue, 16.vii.2011 (16:00), 24.viii.2011 (13:00), 31.viii.2011 (13:00), 12.ix.2011 (12:00), beaten from *Ulmus americana* (5) or *Ulmus parvifolia* (4), P. de Tonnancour (16, CPTO).

### Tribe Smicronychini Seidlitz, 1891

#### Promecotarsus densus

Casey, 1892 new to Alberta

##### Note.

This western North American prairie species is now recorded from Alberta. Nothing is known of the biology of this species.

##### Specimen data.

**Alberta:** Division #1, Onefour, 2.viii.1980, sweeping, G.A.P. Gibson (2, CMNC); Division #1, Cypress Hills Interprovincial Park, 14.viii.1980, sweeping, G.A.P. Gibson (1, CMNC); Division #1, C.F.B. Suffield, 50.628°N, 110.306°W, 28.vii.1994, A.T. Finnamore (6, CMNC).

#### Smicronyx griseus

LeConte, 1876 new to Canada

##### Note.

This native species is distributed in the northeastern United States. Host plants are not known.

##### Specimen data.

**Ontario:** Essex County, Windsor, Ojibway Prairie, 3–7.viii.2001, 12–13.ix.2002, M. Buck & S. Paiero (2, CMNC).

#### Smicronyx lineolatus

Casey, 1892 new to Canada

##### Note.

This native species is distributed in the northeastern United States. Host plants are not known.

##### Specimen data.

**Manitoba:** Junction Highways 21 and 38 N, 49.5626°N, 100.5299°W, 7.vii.2007, tallgrass prairie, sweeping, R. Webster (2, RWC); **Ontario:** Haldimand-Norfolk Region, Delhi-Simcoe Railway Site, 11–14.vii.2001, yellow pans, S. Paiero (1, CMNC).

### Tribe Tychiini Gistel, 1848

#### Lignyodes bischoffi

(Blatchley, 1916) new to New Brunswick

##### Note.

This native eastern North American species is associated with *Fraxinus* (Clark 1980).

##### Specimen data.

**New Brunswick:** Sunbury County, Gilbert Island, 45.8770°N, 66.2954°W, 25.vii–8.viii.2012, 8–21.viii.2012, Lindgren trap in *Tilia americana* canopy, C. Alderson, C. Hughes, & V. Webster (3, RWC); Queens County, Jemseg, 45.8412°N, 66.1195°W, 8–21.viii.2012, Lindgren funnel trap, C. Alderson, C. Hughes, & V. Webster (1, CNMC).

#### Lignyodes horridulus

(Casey, 1892) new to New Brunswick

##### Note.

This native central/eastern North American species is associated with *Fraxinus* (Clark 1980).

##### Specimen data.

**New Brunswick:** York County, New Maryland, Charters Settlement, 45.8395°N, 66.7391°W, 18.vi.2005, UV light, R.P. Webster (1, RWC); Sunbury County, Gilbert Island, 45.8770°N, 66.2954°W, 28.v-12.vi.2012, Lindgren funnel trap, C. Alderson, C. Hughes, & V. Webster (1, CNMC).

#### Tychius liljebladi

Blatchley, 1916 new to Quebec

##### Note.

This widespread western and central native North American species is associated with *Astragalus* spp. (Fabaceae); larvae are in reproductive structures (Anderson 2002). In Canada the species was known from Alberta, Manitoba and Saskatchewan (McNamara 1991); these Quebec records are a significant eastward range extension.

##### Specimen data.

**Quebec:** Gatineau, Aylmer, 18.vii.2004, 22.vii.2004, 28.vi.2005, 8.vii.2005, 9.vii.2011, on flowers of *Astragalus canadensis*, S. Laplante (25, CSLA, 10, CNCI, 2 CMNC); Gatineau, Aylmer, 28.vi.2005, F. Génier (8, CMNC); Gatineau, Aylmer, Queen’s Park, 9.vii.2011, beaten from *Astragalus canadensis*, P. de Tonnancour (26, CPTO).

### Subfamily Bagoinae C.G. Thomson, 1859

#### Pnigodes setosus

LeConte, 1876 new to Canada

##### Note.

This rarely collected native species has been recorded from the central United States north to Montana and South Dakota (O’Brien & Wibmer 1982). It is associated with semi-aquatic habitats or wetlands.

##### Specimen data.

**Alberta:** C.F.B. Suffield, 50.451°N, 110.762°W, 29.vi.1994, pan traps, A.T. Finnamore (3, CMNC).

### Subfamily Baridinae Schönherr, 1836 Tribe Apostasimerini Schönherr, 1844

#### Barinus cribricollis

(LeConte, 1876) new to Canada

##### Note.

This widespread native central USA species is recorded from Canada for the first time. *Barinus* species are associated with sedges in wetlands (Anderson 2002).

##### Specimen data.

**Quebec:** RCM Pierre-De Saurel, Saint-Roch-de-Richelieu, 1.vi.2000, H. Miquet-Sage (3, CHMS); MRC Deux-Montagnes, Oka, Parc d’Oka, 28.v.2002, R. Vigneault (1, CMNC; 1, CRVI); MRC Marguerite-D’Youville, Varennes, 7.vi.2003, C. Chantal (1, CMNC); MRC Vaudreuil-Soulanges, Notre-Dame-de-l’Île-Perrot, 5.vi.2012, swept from *Equisetum*, *Carex* and grasses, P. de Tonnancour (1, CPTO); MRC Marguerite-D’Youville, Verchères, Contrecoeur, 7.vii.2012, swept from *Carex*, sandy bank of Saint-Lawrence River, H. Miquet-Sage (1, CPTO).

#### Sibariops confinis

(LeConte, 1876) new to Canada

##### Note.

This native eastern USA species is recorded from Canada for the first time. *Sibariops* species are associated with sedges in wetlands (Anderson 2002).

##### Specimen data.

**Quebec:** Gatineau, Aylmer, 25.v.2012, swept from Cyperaceae, P. de Tonnancour (3, CPTO).

#### Sibariops confusus

(Boheman, 1836) new to Canada

##### Note.

This widespread native eastern and central USA species is recorded from Canada for the first time. *Sibariops* species are associated with sedges in wetlands (Anderson 2002).

##### Specimen data.

**Quebec:** MRC Le Haut-Richelieu, Saint-Blaise-sur-Richelieu, 19.iv.1980, C. Chantal (1, LEMQ); MRC La Vallée-du-Richelieu, Mont-Saint-Hilaire, 19.v.2004, H. Miquet-Sage (1, CHMS); MRC Vaudreuil-Soulanges, Notre-Dame-de-l’Île-Perrot, 5.vi.2012, swept from *Scirpus* and *Eleocharis*, P. de Tonnancour (1, CPTO).

### Tribe Baridini Schönherr, 1836

#### Cosmobaris scolopacea

Germar, 1819 new to Manitoba

##### Note.

This adventive species is widespread in Canada. It is associated with various Chenopodiaceae (Ciegler 2010).

##### Specimen data.

**Manitoba:** ca. 5 km E. Junction Highways 21 & 345, 49.3849°N, 100.4378°W, 7.vii.2007, sweeping, R.P. Webster (4, RWC).

#### Trichobaris trinotata

(Say, 1832) new to Quebec

##### Note.

This native species, the potato stalk borer, was previously known in Canada only from Ontario. This species is a pest ofvarious Solanaceae; larvae feed in stems (Anderson 2002).

##### Specimen data.

**Quebec:** MRC Deux-Montagnes, Oka, Parc d’Oka, 20.vi.2000 (1, CRVI).

### Tribe Madarini Jekel, 1865

#### Ampeloglypter ampelopsis

(Riley, 1869) new to Quebec

##### Note.

This native species was previously known in Canada only from Ontario. This species is a pest of *Vitis* (grape; Vitaceae); larvae make galls on stems (Anderson 2002), often breaking the vine.

##### Specimen data.

**Quebec:** MRC Vaudreuil-Soulanges, Terrasse-Vaudreuil, 19.v.2011, beaten from *Prunus nigra*, P. de Tonnancour (1, CPTO); MRC Vaudreuil-Soulanges, Notre-Dame-de-l’Île-Perrot, 20 May 2011 (17:00), beaten from *Spiraea x vanhouttei* & *Rubus odoratus*, P. de Tonnancour (2, CPTO); Montreal, Ste-Anne-de-Bellevue, 28.vi.2011, beaten from leaves of *Juglans nigra*, P. de Tonnancour (1, CPTO).

#### Desmoglyptus crenatus

(LeConte, 1876) new to Canada

##### Note.

This rare, native species is known from the northeastern USA: District of Colu-mbia, Maryland, Pennsylvania, and Virginia, and occurs on wild grape, *Vitis* sp. (Vita-ceae) (Blatchley and Leng 1916).

##### Specimen data.

**Ontario:** Essex County, Point Pelee National Park, 11–17 Jul 2003, yellow pan traps in *Opuntia* sp. field, D. Cheung, (1, CMNC), debu00219744.

### Subfamily Ceutorhynchinae Gistel, 1848 Tribe Ceutorhynchini Gistel, 1848

#### Ceutorhynchus hamiltoni

Dietz, 1896 new to Quebec

##### Note.

This native species is widespread along the eastern coastal USA and maritime provinces of Canada (Majka et al. 2007b) on American searocket, *Cakile edentula* Bigelow (Hook.) (Brassicaceae).

##### Specimen data.

**Quebec:** RCM La Haute-Gaspésie, Cap Chat, 21.vii.1954, on *Cakile edentula*, J.E.H. Martin (2, CMNC; 47, CNCI); RCM Bonaventure, New Richmond, 6.viii.1954, on *Cakile edentula*, J.E.H. Martin (5, CNCI).

#### Microplontus campestris

(Gyllenhal, 1837) new to Quebec

##### Note.

This adventive Palaearctic species is associated with *Leucanthemum vulgare* (L.) (Asteraceae) and may help control this invasive weed. This weevil was accidentally introduced into North America, and has been present in Ontario since 1971, or earlier (Anderson and Korotyaev 2004).

##### Specimen data.

**Quebec:** MRC Marguerite-D’Youville, Varennes (Verchères), 30.vi.2008, C. Chantal (1, CCCH).

### Tribe Cnemogonini Colonnelli, 1979

#### Dietzella zimmermanni

(Gyllenhal, 1837) new to New Brunswick

##### Note.

This transcontinental native species is recorded from New Brunswick for the first time. It is associated with *Epilobium* (Ciegler 2010).

##### Specimen data.

**New Brunswick:** Restigouche County, Jacquet River Gorge P.N.A., 47.7491°N, 66.1114°W, 24.vi.2008, R.P. Webster, swept from foliage (1, RWC).

### Tribe Phytobiini Gistel, 1848

#### Parenthis vestitus

Dietz, 1896 new to New Brunswick

##### Note.

This native eastern North American species was previously known in Canada only from Ontario. It is associated with wetlands.

##### Specimen data.

**New Brunswick:** Queens Co., Jemseg, 45.8412°N, 66.1195°W, 25.vii-8.viii.2012, C. Alderson, C. Hughes, & V. Webster, Lindgren trap (1, RWC); Sunbury County, Gilbert Island, 45.8770°N, 66.2954°W, 18–28.V.2012, 25.V–12.vi.2012, 11–25.vii.2012, Lindgren trap under *Juglans cinerea*, C. Alderson, C. Hughes & V. Webster (1, CMNC; 5, RWC); Sunbury County, Gilbert Island, 45.8770°N, 66.2954°W, 21.viii-7.ix.2012, C. Hughes & K. Van Rooyen (1, CMNC).

#### Pelenomus squamosus

LeConte, 1876 new to New Brunswick

##### Note.

This transcontinental North American species is recorded from the Maritime Provinces for the first time. It is associated with wetlands.

##### Specimen data.

**New Brunswick:** Queens County, Jemseg, 45.8412°N, 66.1195°W, 14–28.v.2012, Lindgren funnel, C. Alderson, C. Hughes, & V. Webster (1, RWC); Restigouche County, Wild Goose Lake, 420 m elev., 47.8540°N, 66.3219°W, 7.vi.2012, treading *Carex* & grasses, R. Webster & M. Turgeon (1, RWC); Sunbury County, Gilbert Island, 45.8770°N, 66.2954°W, 11–25.vii.2012, Lindgren trap, C. Alderson, C. Hughes & V. Webster (1, CMNC).

### Tribe Scleropterini Schultze, 1902

#### Asperosoma echinatum

(Fall, 1917) new to Ontario

##### Note.

**Figure 1.**
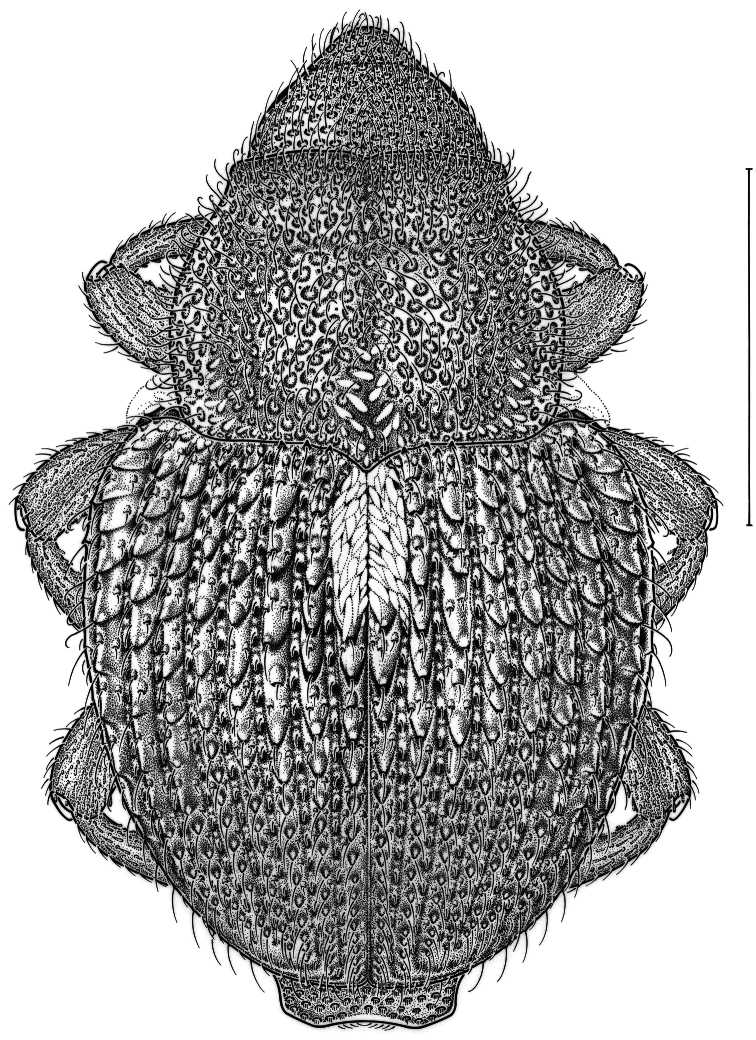
Dorsal habitus drawing of *Asperosoma echinatum* (Fall, 1917), a species to-date only known from Canada. Scale line = 1mm.

This species (Fig. 1) is associated with the native grassland forb *Heuchera richardsoni* R. Br. (Saxifragiaceae) (Fall 1917) and was previously known only from Manitoba (McNamara 1991). This species is at present a Canadian endemic, although it may also exist in USA. Targeted collecting efforts at other Ontario sites have not yielded additional specimens.

##### Specimen data.

**Ontario:** Essex Co., Windsor, Burnt Prairie, v.2001, S. Paiero, CNC COLEOPT #04-5422 (1, CMNC).

### Subfamily Conoderinae Schönherr, 1833 Tribe Lechriopini Lacordaire, 1865

#### Psomus armatus

Dietz, 1891 new to New Brunswick

##### Note.

This native eastern North American species is recorded from the Maritime Provinces for the first time. It is associated with forest habitats, and has been recorded on sprouts of white ash, *Fraxinus americanus* L. (Blatchley and Leng 1916).

##### Specimen data.

**New Brunswick:** Carleton County, Jackson Falls, Bell Forest, 46.2200°N, 67.7231°W, 3–17.vii.2012, Lindgren trap in *Tilia americana* canopy, C. Alderson & V. Webster(1, RWC); Queens County, Jemseg, 45.8412°N, 66.1195°W, 28.vi–10.vii.2012, Lindgren funnel trap, C. Alderson &V. Webster (1, RWC).

### Tribe Zygopini Lacordaire, 1865

#### Cylindrocopturus furnissi

Buchanan, 1940 new to Canada

##### Note.

This native species, known in USA as the Douglas-fir twig weevil, was recorded from California, Oregon and Washington by O’Brien and Wibmer (1982). It is a minor pest of shoots of weakened *Pseudotsuga* spp. (Pinaceae).

##### Specimen data.

**British Columbia:** Kootenays Region, Wynndel (2 mi. N.), 28.vi.–3.vii.1980, R. Anderson (1, CMNC).

#### Cylindrocopturus quercus

(Say, 1832) new to Canada

##### Note.

This native species was recorded from eastern USA by O’Brien and Wibmer (1982). Adults of this species breed in ragweed, *Ambrosia artemesiifolia* L. (Piper 1977).

##### Specimen data.

**Quebec:** Montreal, (1, CNCI); Ormstown, 29.vi.1978, E.J. Kiteley (1, CNCI); MRC Vaudreuil-Soulanges, Notre-Dame-de-l’Île-Perrot, 19.vi.2011 (17:00), 12.viii.2012 (17:00), swept from *Erigeron* sp., beaten from *Asclepias syriaca*, P. de Tonnancour (3, CPTO).

### Subfamily Cossoninae Schönherr, 1825 Tribe Dryotribini LeConte, 1876

#### Caulophilus dubius

(Horn, 1873) new to Canada

##### Note.

This native species was recorded from eastern USA north to Illinois, Michigan, New York, Ohio and Pennsylvania by O’Brien and Wibmer (1982). Adults are found beneath the dead tree bark (Blatchley and Leng 1916).

##### Specimen data.

**Quebec:** Montreal, Sainte-Anne-de-Bellevue, Morgan Arboretum, yellow pan traps, 25–29.vi.2001, J. Forrest (1, LEMQ).

### Tribe Onycholipini Wollaston, 1873

#### Pseudopentarthrum parvicollis

(Casey, 1892) new to Canada

##### Note.

This native species is widespread in the eastern USA from TX north to NY. It is associated with decaying wood, particularly old tree holes.

##### Specimen data.

**Ontario:** Kent County, Rondeau Provincial Park, Spicebush Trail, 42°18'09"N, 81°51'06"W, 16–29 Jul 2003, S.A. Marshall, Malaise Trap in Carolinian forest (1; CMNC), debu01123692.

### Tribe Rhyncolini Gistel, 1848

#### Rhyncolus knowltoni

(Thatcher, 1940) new to Alberta, and Saskatchewan

##### Note.

This native western North American species was previously only known in Canada from Manitoba. It has been associated with *Populus tremuloides* Michx.

##### Specimen data.

**Alberta:** Southern Alberta, Medicine Hat, viii.1980, pan trap, G.A.P. Gibson, (1; CMNC); Division #2, 0.5 km E. Writing-on-Stone Provincial Park, 6–15.vi.1981, pan traps, D. McCorquodale (1; CMNC); Division #1, Cypress Hills Interprovincial Park, 4 km S. Elkwater, 22.vi-19.viii.1988, 1400m, S. & J. Peck, fir-pine-aspen forest, FIT (1; CMNC); **Saskatchewan:** Maple Creek No. 111,Cypress Hills Interprovincial Park, Center Block, Boiler Creek aspen forest, 21.vi-19.viii.1988, FIT 1200m, S. & J. Peck (1; CMNC).

#### Rhyncolus macrops

Buchanan, 1946 new to New Brunswick

##### Note.

This transcontinental native North American species is recorded from New Brunswick for the first time. Adults are associated with a variety of dead coniferous trees (Pinales) (Buchanan 1946).

##### Specimen data.

**New Brunswick:** Carleton County, Jackson Falls, Bell Forest, 46.2200°N, 67.7231°W, 7.vi.2007, 27.vi.2008 under spruce bark & in Lindgren trap, R. Webster (2, RWC); Sunbury County, Acadia Research Forest, 45.9866°N, 66.3841°W, 19–25.v.2009, 25.v–2.vi.2009, 16–24.vi.2009, red spruce forest, Lindgren traps, R. Webster & M.A. Giguère (3, RWC); York County, Charters Settlement, 45.8380°N, 66.7300°W, 6.v.2004, under bark, R. Webster (1, RWC).

### Subfamily Cryptorhynchinae Schönherr, 1825 Tribe Cryptorhynchini Schönherr, 1825

#### Acalles carinatus

LeConte, 1876 new to Quebec

##### Note.

Label data from many specimens from throughout its range in eastern USA and southern Ontario indicates that this native species occurs commonly in hardwood forest leaf litter.

##### Specimen data.

**Quebec:** MRC Deux-Montagnes, Oka, Parc d’Oka, 11.vi.1995, R. Vigneault (1, CRVI); MRC La Vallée-du-Richelieu, Mont-Saint-Hilaire, yellow pan traps, 11–18.vi.2001, E. Fast (1, CMNC); MRC La Vallée-du-Richelieu, Mont-Saint-Hilaire, beach-sugar maple forest, yellow pan traps, 21–28.v.2001, E. Fast (1, LEMQ); same except: 16–23.vii.2001 (1, LEMQ); Montreal, Sainte-Anne-de-Bellevue, Morgan Arboretum, 10–15.vi., 15–20.vi., 20–25.vi., 3–9.vii., 20–26.viii.2001, J. Forrest, yellow pan traps in ash-sugar maple forest (6, LEMQ).

#### Cryptorhynchus tristis

LeConte, 1876 new to Canada

##### Note.

This widespread native eastern USA species is said to feed on leaves of *Quercus coccinea* Wang. (Fagaceae, scarlet oak) at night and larvae develop under the bark (Anderson 2008).

##### Specimen data.

**Quebec:** RCM Brome-Missisquoi, Saint-Armand, 12.vii.2008, P. Bélanger, UV light (1, LEMQ).

#### Tyloderma foveolatum

(Say, 1832) new to Manitoba

##### Note.

This widespread native eastern USA and eastern southern Canadian species has been associated with *Oenothera biennis* L. (Onagraceae), a native ruderal plant (Wibmer 1981).

##### Specimen data.

**Manitoba:** Eastern Manitoba, Sandilands Provincial Forest, Marchand (10–12 km E.), 10–12.vi.1987, H. & A. Howden (1, CMNC); Spruce Woods Provincial Park, Glenboro (10–15 km W.), 17.vi.1987, H. & A. Howden (3, CMNC).

### Subfamily Cyclominae Schönherr, 1826 Tribe Listroderini LeConte, 1876

#### Listronotus maculicollis

(Kirby, 1837) new to Manitoba

##### Note.

This transcontinental North American native species is recorded from Manitoba for the first time. It is associated with wetlands.

##### Specimen data.

**Manitoba:** Aweme at Assiniboine River, 49.673°N, 99.565°W, 11.vii.2003, R.P. Webster (1, RWC).

#### Listronotus punctiger

LeConte, 1876 new to Manitoba

##### Note.

This western North Americans native pecies is recorded from Manitoba for the first time. It is associated with wetlands.

##### Specimen data.

**Manitoba:** near Junction Highways 21 & 543 N, 49.6705°N, 100.4646°W, 6.vii.2007, sweeping, R.P. Webster (1, RWC).

### Subfamily Entiminae Schönherr, 1823 Tribe Cyphicerini Lacordaire, 1863

#### Cyrtepistomus castaneus

(Roelofs, 1873) new to Quebec

##### Note.

This adventive Palaearctic species is widespread in the eastern USA into Ontario and is known in USA as the Asiatic oak weevil; it can be extremely common locally (Anderson 2002), and acts as a minor defoliator of broadleaved trees.

##### Specimen data.

**Quebec:** RCM La Côte-de-Gaspé, Gaspé, 27.viii.1966, W. Boyle (1, LEMQ).

### Tribe Naupactini Gistel, 1848

#### Atrichonotus taeniatulus

(Berg, 1881) new to Canada

##### Note.

This adventive species, the adults of which feed on the roots and foliage of a variety of host plants (although most frequently on Fabaceae), was known previously in North America from southeastern USA west to Texas (Anderson 2002). This species can be an important pest of alfalfa (*Medicago sativa* L., Fabaceae).

##### Specimen data.

**Quebec:** MRC La Vallée-du-Richelieu, Mont-Saint-Hilaire, 25.v.1999, H. Miquet-Sage (1, CHMS).

### Tribe Otiorhynchini Schönherr, 1826

#### Otiorhynchus ligustici

(Linnaeus, 1758) new to Quebec

##### Note.

This adventive Palaearctic species was known in Canada only from Ontario (Bright and Bouchard 2008). Also known as the alfalfa snout beetle this species is a major pest of alfalfa.

##### Specimen data.

**Quebec:** MRC Beauharnois-Salaberry, Salaberry-de-Valleyfield, 13.vi.2012, on grasses under *Salix*, *Populus* and *Vitis riparia*, Y. Racine (1, CPTO).

### Subfamily Lixinae Schönherr, 1823 Tribe Lixini Schönherr, 1823

#### Larinus planus

(Fabricius, 1792) new to Quebec

##### Note.

This adventive Palaearctic species was introduced to North America for the biological control of Canada thistle, *Cirsium arvense* (L.) Scop. (Asteraceae) (Anderson 2002). It is present in Alberta, British Columbia, Nova Scotia and Ontario.

##### Specimen data.

**Quebec:** MRC Marguerite-D’Youville, Varennes (Verchères), 25.viii.2001, 6.ix.2001, 16.viii.2004, 12.vi.2006, 13.vi.2006, C. Chantal (8, CCCH); MRC Vaudreuil-Soulanges, Notre-Dame-de-l’Île-Perrot, 20.vi.2009, 21.vi.2009, 30.viii.2009, 30.viii.2012, all on *Cirsium arvense*, P. de Tonnancour (6, CPTO); Montreal, Sainte-Anne-de-Bellevue, 25.vi.2010, 7.vi., 11.vi.2011, on *Cirsium arvense*, P. de Tonnancour (12, CPTO).

### Subfamily Mesoptiliinae Lacordaire, 1863 Tribe Magdalidini Pascoe, 1870

#### Magdalis inconspicua

Horn, 1873 new to New Brunswick

##### Note.

This native eastern North American species is recorded from the Maritime Provinces for the first time. It is associated with forest habitats.

##### Specimen data.

**New Brunswick:** Sunbury County, Gilbert Island, 45.8770°N, 66.2954°W, 29.vi.–11.vii.2012, Lindgren traps in canopy of *Juglans cinerea* & *Tilia americana*, and under *Tilia americana*, C. Alderson & V. Webster (2, AFC; 1, CMNC; 1, NBM; 7, RWC); Carleton County, Jackson Falls, Bell Forest, 46.2200°N, 67.7231°W, 7–21.vi.2012, Lindgren traps in *Tilia americana* canopy, C. Alderson & V. Webster (1, CMNC; 1, RWC).

#### Magdalis salicis

Horn, 1873 new to New Brunswick

##### Note.

This native eastern North American species is recorded from New Brunswick for the first time. It is associated with forest habitats.

##### Specimen data.

**New Brunswick:** Queens County, Grand Lake Meadows PNA, 45.8227°N, 66.1209°W, 21.vi-5.vii.2011, Lindgren trap in canopy, M. Roy & V. Webster (1, RWC); Sunbury County, Gilbert Island, 45.8770°N, 66.2954°W, 12.vii.2012, sweeping, R.P. Webster (1, RWC).

### Subfamily Molytinae Schönherr, 1823 Tribe Conotrachelini Jekel, 1865

#### Microhyus setiger

LeConte, 1876 new to Quebec

##### Note.

Adults of this widespread native eastern USA (and into Ontario) species have been associated with dead *Fagus* (Fagaceae, beech) (Anderson 2002).

##### Specimen data.

**Quebec:** MRC Deux-Montagnes, Oka, Parc d’Oka, 30.v.1995, R. Vigneault (1, CRVI); Brome-Missisquoi, Saint-Armand, 9.vi.2003, Claude Chantal (1, CCCH); MRC Marguerite-D’Youville, Varennes (Verchères), 6.vi.2011, C. Chantal (1, CCCH).

### Subfamily Scolytinae Latreille, 1804

The Scolytinae, or bark beetles are a distinctive and relatively well-known subfamily that includes many forest pests. Scolytinae have been a focus of adventive forest pest trapping surveys by the Canadian Food Inspection Agency, the Canadian Forest Service and others. The taxonomy and distribution of species that are not readily captured in traps, or attack smaller diameter stems remain less well-known.

### Tribe Dryocoetini Lindemann, 1877

#### Dryocoetes autographus

(Ratzeburg, 1837) new to Nova Scotia

##### Note.

This widespread native species, known from all other provinces and two territories, attacks the lower parts of dead and dying conifers. The absence of records from Nova Scotia seems to be an oversight.

##### Specimen data.

**Nova Scotia:** Colchester Co., Portapique, 45.405°N, 63.704°W, 26.vii.1927, C.A. Frost (1, CNCI), CNC Diptera 126927; Kejimkujik National Park, 44.386°N, 65.293°W, 17.vii.1970, ex. *Picea glauca*, D.E. Bright (2, CNCI), CNC Diptera 127810, 127811; St. Ann’s Gut, 46.217°N, 60.616°W, 3.viii.1970, ex. *Picea*, D.E. Bright (2, CNCI), CNC Diptera 127830, 127831; White point, 46.883°N, 60.363°W, 23.vi.1983, Y. Bousquet (1, CNCI), CNC Diptera 128020, 128021, 128022; Cape Breton Highlands National Park, Lone Shieling, 46.897°N, 60.783°W, 1.vii.1983, R. Vockeroth, L. Lesage, Y. Bousquet (2, CNCI), CNC Diptera 128037, 128038, 128072; Cape Breton Highlands National Park, Jack Pine Trail, 46.779°N, 60.333°W, 22.vii.1983, D.E. & J.E. Bright (1, CNCI), CNC Diptera 128028; Halifax, Point Pleasant Park, 46.822°N, 60.799°W, 13.vi.–5.vii.1990, S. Robertson & G Harding (4, CNCI), CNC Diptera 127944, 127945, 127946, 127947.

#### Dryocoetes caryi

Hopkins, 1915 new to Prince Edward Island

##### Note.

This rarely collected native species typically inhabits small, stressed *Picea* spp. (Pinaceae) trees and is known from across Canada (Alberta, British Columbia, New Brunswick, Nova Scotia, and Quebec) Bright 1976, Webster et al. 2012).

##### Specimen data.

**Prince Edward Island**, Queens Co., Charlottetown, 23.vi.–7.vii.2000, funnel trap, CFIA (1, CNCI).

#### Lymantor alaskanus

Wood, 1978 new to Canada

##### Note.

This native species was only known from the type series, collected in 1978 near Fairbanks Alaska. The Alberta specimens mentioned here represent a significant extension of the known range to the south and east. Both the type series and all specimens reported here were captured in CFIA traps baited with ipsenol lure.

##### Specimen data.

**Alberta:** RM (Regional Municipality) of Wood Buffalo, 56.733°N, 111.384°W, 7.vii.2005, funnel trap with ipsenol, CFIA (3, CNCI), CNC COLEO 00106296, 00106297; RM of Wood Buffalo, 56.733°N, 111.384°W, 28.iv.–29.ix.2005, funnel trap (2, CFIA), CNCI, CNC COLEO 00106298.

### Tribe Hylastini LeConte, 1876

#### Hylastes macer

LeConte, 1868 new to Alberta

##### Note.

This western species feeds mainly on *Pinus* spp. (Pinaceae), and was already known from nearby parts of British Columbia (Bright 1976).

##### Specimen data.

**Alberta:** Calgary, 51.042°N, 114.078°W, 11.vi.1944, in flight, E.J. Kitely (1, CNCI), CNC Diptera 129000.

#### Hylastes opacus

Erichson, 1836 new data on first Canadian and Quebec records

##### Note.

These records of this adventive, *Pinus*-feeding Palaearctic species (also known from New Brunswick, Webster et al. 2012) were reported by Bright and Skidmore (1997) without specimen data.

##### Specimen data.

**Ontario:** Elgin Co., Port Bruce, 42.650°N, 81.017°W, 19.iv.1995, J. Hale (2, CNCI), CNC COLEO 00105928, 00105929; **Quebec:** Montreal, 45.500°N, 73.600°W, 6.vi.1997, D. Couture (2, CNCI), CNC COLEO 00105926, 00105927.

### Tribe Hylurgini Gistel, 1848

#### Dendroctonus ponderosae

Hopkins, 1902 new to Alberta

##### Note.

The native, *Pinus*-feeding mountain pine beetle has not previously been reported from Alberta in the taxonomic literature despite well-studied, costly outbreaks there. Specimens listed here document the oldest CNCI material from Canada outside British Columbia.

##### Specimen data.

**Alberta:** Waterton Lakes National Park, Summit-Carthew Lakes Trail, 49.033°N, 113.984°W, 17.vi.1980, J.M Campbell & D.E. Bright (6, CNCI), CNC Diptera 130912–130917; Waterton Lakes National Park, km 3 Chief Mountain Highway, 17.vi.1980, J.M. Campbell (1, CNCI), CNC Diptera 130911; Waterton Lakes National Park, Red Rock Canyon, 49.133°N, 113.018°W, 16.vii.1980, ex. *Pinus contorta*, D.E. Bright(1, CNCI), CNC Diptera 130910; Waterton Lakes National Park, km 9 Chief Mountain Highway, 24.vii.1980, ex. *Pinus contorta*, D.E. Bright (3, CNCI), CNC Diptera 130918–130920; Waterton Lakes National Park, Cameron Lake, 49.017°N, 114.067°W, 30.vii.1980, D.E. Bright (4, CNCI), CNC Diptera 130902–130905; Waterton Lakes National Park, Belly River, 49.767°N, 113.034°W, 30.vii.1980, D.E. Bright (4, CNCI), CNC Diptera 130906–130909.

#### Dendroctonus simplex

LeConte, 1868 new to Yukon and Nunavut: not known from Northwest Territories

##### Note.

The native eastern larch beetle is reported from all ten provinces, and Northwest Territories (Bright 1976). With the separation of Nunavut from Northwest Territories, the single Northwest Territories record should become a Nunavut record.

##### Specimen data.

**Nunavut:** Keewatin, Padlei, 61.933°N, 96.650°W, 27.vii.1950, R.E. Duckworth (1, CNCI), CNC COLEO 00100743; **Yukon:** Km 382, Dempster Highway, 66.386°N, 136.317°W, 23.vi.1981, D.E. Bright (1, CNCI), CNC Diptera 132434.

#### Tomicus piniperda

(Linnaeus, 1758) new data on first Canadian record, and first Quebec record

##### Note.

These records of this adventive Palaearctic species, the pine shoot beetle, were reported by Bright and Skidmore (1997) without specimen data.

##### Specimen data.

**Ontario:** Haldimand Co., Dunnville, 42.904°N, 79.618°W, iv.1993, Agriculture Canada (8, CNCI), CNC Diptera 128768 to 128775; Haldimand Co., Dunnville, 42.904°N, 79.618°W, 23.vi.1993, ex. bole of *Pinus sylvestris*, D.E. Bright (12, CNCI), CNC Diptera 128776 to 128782; **Quebec:** Gatineau,Aylmer, 45.400°N, 75.817°W, 9.vii.1993, ex. bole *Pinus sylvestris*, D.E. Bright (1, CNCI), CNC Diptera 128783.

### Tribe Ipini Bedel, 1888

#### Ips perroti

Swaine, 1915 new to Nova Scotia

##### Note.

This native species breeds in thin-barked *Pinus* spp. stems, and is known from Alberta, British Columbia, Manitoba, New Brunswick, Ontario, Quebec, and Saskatchewan.

##### Specimen data.

**Nova Scotia:** Cape Breton, Bras d’Or, 46.250°N, 60.282°W, 1–21.vi.2000, ex. funnel trap, CFIA (1, CNCI), CNC COLEO 00105995.

### Tribe Micracidini LeConte, 1876

#### Hylocurus rudis

(LeConte, 1876) new to Canada

##### Note.

This native species breeds in weakened or dead small diameter stems of hardwood trees. Its apparent limitation to southern Ontario and Quebec is probably due to climate, given that it is also known from Michigan, Ohio and Pennsylvania (Wood and Bright 1992).

##### Specimen data.

**Ontario:** Essex Co., Pt. Pelee National Park, Visitor’s Centre, 22–29.v.2000, O. Lonsdale (1, DEBU), debu01000657; Essex Co., Middle Island, 40°41'N, 82°41'W, 4.vii.2000, ex. yellow pans etc., Paiero, Marshall, & Cheung (4, DEBU), debu00221910, debu00222016, debu00221976, debu00221526; Essex Co., Middle Island, 40°41'N, 82°41'W, 11.vi.2003, S.A. Marshall (1, DEBU), debu00221012; Essex Co., Pt. Pelee National Park, The Tip, 17.vi.2003, H. Carscadden (1, DEBU), debu00219553; Halton, Oakville, 43.450°N, 79.683°W, 9–23.vi.2008, ex. funnel trap, CFIA (1, CNCI), CNC COLEO 00105968; **Quebec:** MRC Deux-Montagnes, Oka, Parc d’Oka, Deux-Montagnes, 27.iv.1997, reared ex. *Carya ovata* R. Vigneault, (1, CRVI).

#### Micracis suturalis

LeConte, 1868 new to Ontario

##### Note.

This native species breeds in *Cercis* spp. (Fabaceae), *Juglans* spp. (Juglandaceae), and other broadleaved trees (Bright 1976), and is already known from Quebec (Chantal 1992).

##### Specimen data.

**Ontario:** Kent Co., Rondeau Provincial Park, 1.vi.1982, D.E. Bright (1, CNCI), CNC COLEO 00155768.

#### Pseudothysanoes rigidus

(LeConte, 1876) new data on first Ontario record

##### Note.

This native species, known from USA, breeds in *Tilia* sp. (Malvaceae), and has been previously reported from Ontario without additional data (Bright 1976).

##### Specimen data.

**Ontario:** RM Halton, Burlington, Sheldon Creek Woodlot, 43.396°N, 79.775°W, 1–14.vi.2007, ex. funnel trap, CFIA (1, CNCI), CNC COLEO 00106106.

### Tribe Phloeosinini Nüsslin, 1912

#### Phloeosinus pini

Swaine, 1915 new to Ontario

##### Note.

This widespread (Alberta, British Columbia, Manitoba, Quebec, Northwest Territories, Nova Scotia, Yukon), but infrequently collected native species breeds in *Pinus* and *Picea* (Bright 1976).

##### Specimen data.

**Ontario:** Lennox and Addington Co., Napanee, 44.267°N, 76.971°W, 20.v.2004, ex. funnel trap, CFIA (1, CNCI).

### Tribe Phloeotribini Chapuis, 1869

#### Phloeotribus liminaris

(Harris, 1852) new to Saskatchewan

##### Note.

The peach bark beetle breeds in *Prunus* spp., is native and already known from neighbouring Manitoba, and also Ontario, Quebec, New Brunswick, and Nova Scotia (Bright 1976, Majka et al. 2007).

##### Specimen data.

**Saskatchewan:** Saskatoon, Avenue K. S., vi.2011. J. Boone (1, City of Saskatoon).

#### Phloetribus piceae

Swaine, 1911 new to Yukon

##### Note.

This infrequently collected boreal species breeds in *Picea* spp (Bright 1976), and was known from British Columbia, Manitoba, Ontario, Quebec, New Brunswick, Nova Scotia, and Northwest Territories.

##### Specimen data.

**Yukon:** Km 72, Dempster Highway, 64.53°N, 138.231°W, 20.vi.1981, ex. *Picea glauca*, D.E. Bright (6, CNCI), CNC COLEO 00104642, CNC Diptera 130333–130336.

#### Phloeotribus scabricollis

(Hopkins, 1916) new to Canada

##### Note.

This rarely collected species breeds in *Ptelea trifoliata* L. (Rutaceae) and *Staphylea trifolia* L. (Staphyleaceae) (Wood 1982).

##### Specimen data.

**Ontario:** Essex Co., Pelee Island, 11.vi.2003, J. Ambrose (1, DEBU), 00137345.

### Tribe Scolytini Latreille, 1804

#### Scolytus multistriatus

(Marsham, 1802) new data on first Alberta record

##### Note.

This record of this adventive Palaearctic species was reported by Bright and Skidmore (1997) without specimen data. *Scolytus multistriatus* is a pest of *Ulmus* spp., and also known from British Columbia, Manitoba, Ontario, Nova Scotia, Quebec, and Saskatchewan.

##### Specimen data.

**Alberta:** Calgary, 51.050°N, 114.084°W, 19.vii.1994, T. Reichardt (2, CNCI), CNC Diptera 134316 to 134317; Edmonton, 53.554°N, 113.406°W, 6.vii.–14.viii.1995, C. Brososky (1, CNCI), CNC Diptera 134314.

#### Scolytus oregoni

Blackman, 1934 new to Canada

##### Note.

This infrequently collected native species breeds in *Pseudotsuga* sp. in USA (Wood 1982).

##### Specimen data.

**British Columbia:** Vancouver Island, Victoria, 48.541°N, 123.469°W, 11–25.viii.2009, ex. funnel trap, CFIA, (3, CNCI).

#### Scolytus piceae

(Swaine, 1910) new to Newfoundland

##### Note.

This infrequently collected native species breeds mainly in *Picea* spp. (Bright 1976). It is otherwise known from all other provinces and territories except for Prince Edward Island.

##### Specimen data.

**Newfoundland:** Humber District–Corner Brook, 12 mi NE Deer Lake, 49.318°N, 57.212°W, 23.vii.1970, ex. *Picea mariana*, D.E. Bright (1, CNCI), CNC Diptera 133720.

#### Scolytus rugulosus

(Müller, 1818) new data on first Canadian record

##### Note.

This adventive Palaearctic species was apparently reported from Canada in Ontario by Chamberlin (1939) without specimen data. It breeds in *Malus*, *Prunus* and *Pyrus* trees.

##### Specimen data.

**Ontario:** Prince Edward Co., 2.vii.1917, J.F. Brimley (1, CNCI), CNC Diptera 133735.

#### Scolytus schevyrewi

Semenov Tjan-Shansky, 1902 new to Alberta, Saskatchewan, Manitoba, and Ontario

##### Note.

This adventive Palaearctic species is known from British Columbia (Humble et al. 2010), and breeds in elms, particularly damaging the adventive *Ulmus pumila* (Ulmaceae).

##### Specimen data.

**Alberta:** Medicine Hat, Gershaw Avenue, 1–30.ix.2006, ex. funnel trap, CFIA (1, CNCI), CNC COLEO 00105817; **Manitoba:** RM De Salaberry Otterburne, 49.973°N, 97.052°W, 2007, ex. funnel trap, CFIA, (2, CNCI), CNC COLEO 00106339, 00106340; **Ontario:** RM Peel, Mississauga, 43.711°N, 79.722°W, 6–19.viii.2008, ex. funnel trap, CFIA (1 CNCI); RM Hamilton, 43.269°N, 79.83°W, 29.v.–11.vi.2012, ex. funnel trap, CFIA (1, no voucher retained); Lambton Co., Sarnia, 43.986°N, 82.410°W, 11–25.vi., 30.vi.–11.vii.2012, ex. funnel trap, CFIA (2, CNCI); **Saskatchewan:** Maple Creek, 49.917°N, 109.484°W, 21.iv.2007, ex. funnel trap, CFIA (1, CNCI), CNC COLEO 00105818; Regina, v.–vii.2007, ex. funnel trap, CFIA (1, CNCI), CNC COLEO 00106339 (specimens also examined from Assiniboia, Eston, Estevan, Moose Jaw, Shaunavon, Weyburn, Yorkton).

### Tribe Xyleborini LeConte, 1876

Members of this tribe are obligate symbionts of fungi, which they introduce and cultivate in the xylem of their woody hosts.

#### Euwallacea validus

(Eichhoff, 1875) new to Canada

##### Note.

This adventive species has been present in North America since 1976 and has since spread within the eastern USA (Rabaglia et al. 2006). *Euwallacea validus* breeds in a variety of broadleaved and conifer trees, and its pest-status remains unclear.

##### Specimen data.

**Ontario:** Essex Co., Windsor, Bloomfield & Watkins, 42.291°N, 83.076°W, 4.ix.2004, ex. *Ailanthus altissima*, E. Czerwinski (2, CNCI), CNC COLEO 00105866, 00105867; Niagara, Douglastown, 42.974°N, 79.018°W, 22.xi.2005, ex. *Pinus sylvestris*, L. Tucker(1, CNCI), CNC COLEO 00106198.

#### Xyleborinus attenuatus

(Blandford, 1894) new to Nova Scotia, Ontario and Quebec

##### Note.

This adventive species, which was until recently known mainly by the synonym *Xyleborinus alni* Niisima, is a recent arrival in North America (Mudge et al. 2001). It was found soon after initial detection in much of NE and NW USA and British Columbia and Prince Edward Island. *Xyleborinus attenuatus* feeds on woody angiosperms, and its pest-status remains unclear.

##### Specimen data.

**Nova Scotia:** Halifax, 44.738°N, 63.546°W, 29.v.–12.vi.2007, ex. funnel trap, CFIA (2, CNCI), CNC COLEO 00106125, 00106126; **Ontario:** Middlesex Co., London, 42.983°N, 81.233°W, 27.iv.1998, ex. funnel trap, CFIA (2, CNCI), CNC COLEO 00106144, 00106145; **Quebec:** Sherbrooke, 45.417°N, 71.900°W, 20.v.–2.vi.2009, ex. funnel trap, CFIA (1, CNCI), CNC COLEO 00106128.

#### Xyleborus affinis

Eichhoff, 1868 new to Quebec

##### Note.

This apparently native species is already known from neighbouring Ontario and New York State (Rabaglia et al. 2006), and breeds in deciduous trees.

##### Specimen data.

**Quebec:** MRC Deux-Montagnes, Oka, Parc d’Oka, Lac de la Sauvagine, 2.xi.2002, R. Vigneault (2 CNCI; 23, CRVI), CNC Diptera 125448, 125449.

#### Xyleborus celsus

Eichhoff, 1868 new to Canada

##### Note.

This native species breeds in *Carya* spp. (Juglandaceae) in the USA (Rabaglia et al. 2006). In Canda these trees occur in only southern parts of Ontario and Quebec.

##### Specimen data.

**Ontario:** Kent Co., Rondeau Provincial Park, 42.329°N, 81.846°W, 9.vi.1980, H. Goulet (1, CNCI), CNC Diptera 125451; Kent Co., Rondeau Provincial Park, Visitor’s Centre, 42.781°N, 81.844°W, 3.vii.2003, S.M. Paiero (1, DEBU), debu01119222; Middlesex Co., London, 43.090°N, 81.187°W, 13.viii.2006, ex. sticky trap, K. Nystrom (1, CNCI; 1, GLFC), CNC COLEO 00106117.

#### Xyleborus ferrugineus

(Fabricius, 1801) new to Canada

##### Note.

This apparently native species, feeds in a wide variety of woody plants, and is known in the USA from nearby Michigan, New York, Ohio and Pennsylvania, and is also present as an adventive on other continents (Rabaglia et al. 2006).

##### Specimen data.

**Ontario:** Kent Co., Rondeau Provincial Park, 11–25.v.1985, flight intercept trap in maple-beech forest, L. Lesage and A. Woodliffe, (1, CNCI).

#### Xylosandrus crassiusculus

(Motschulsky 1866) new to Canada

##### Note.

This east-Asian species is known from throughout the eastern USA (Rabaglia et al. 2006), including nearby Ohio (Lightle et al. 2007) and Michigan (Cognato et al. 2009), from as far north as 45.350°N. This species breeds in many broadleaved woody plants and is a pest in the USA in apparently healthy nursery material and fruit trees. (Kovach and Gorsuch 1985, Oliver and Mannion 2001)

##### Specimen data.

**Ontario:** Elgin Co., 42.826°N, 81.288°W, 11–25.vi., 12–26.vii., 10–24.viii., 18.ix.–2.x.2012, ex. funnel trap, CFIA (27, CNCI).

#### Xylosandrus germanus

(Blandford, 1894) new data on first Quebec record

##### Note.

This adventive Palearctic species was first reported from Quebec by Bright and Skidmore (2002) without reference to specimens. The following is to document these first-known captures of this species in Quebec. *Xylosandrus germanus* feeds in both broad leaved and conifer trees.

##### Specimen data.

**Quebec:** MRC Longueuil, Saint-Bruno-de-Montarville, Mont St-Bruno, 45.55°N, 73.316°W, 30.v.–5.vi., 5–12.vi., 12–19.vi., 19–26.vi., 26.vi.–2.vii., 2–12.vii., 12–17.vii., 17–24.vii., 24.vii.–1.viii., 1–8.viii.2000, G. Pelletier (18 CNCI), CNC Diptera 128373 to 128391.

## Discussion

McNamara (1991) provided a comprehensive list of Curculionoidea known from Canada and its provinces at that time. Noteworthy additions to our knowledge of the Canadian weevil fauna in the last 20 years include the works published by Bright (1993, 1994), Bouchard et al. (2005), Bright and Skidmore (1997), Anderson (1997), Chantal (1998), Anderson (2002), Anderson and Korotyaev (2004), Rabaglia et al. (2006), Majka and Anderson (2007), Majka et al. (2007a–c), Bright and Bouchard (2008), Humble et al. (2010), Klimaszewski et al. (2010), De Clerck-Floate and Cárcamo (2011), Webster et al. (2012), Bouchard et al. (2012), Humble and Hueppelsheuser (2012) and Looney et al. (2012). Beyond these, we list 24 species new to Canada, in ten cases also representing new records at the generic level. We have also added 59 species new to 12 provinces and territories. These records include 10 pest species and six species introduced elsewhere as biological control agents. Some are new records of adventive species expanding their range, others may be northward expansions of species common in the USA, and many fill gaps within the patchy known distributions of infrequently collected native species.

The present review of collections material for new distributional records was undertaken in anticipation of the new checklist of Canadian Coleoptera (Bousquet et. al 2013). Except for the maratimes provinces, which are now receiving increased faunistic research (e.g. Majka and Anderson (2007), Majka et al. (2007a–c), Webster et al. (2012)), our understanding of most other beetle families would also benefit from such a review. It is also probable that further undocumented curculionoid first records remain to be gleaned from material at Canadian insect collections which we were unable to include in this study.

The range extension for *Asperosoma echinatum* from Manitoba into southern Ontario is partidularly interesting. It is very uncommon for species to be endemic to Canada but this is an example where both the genus and species are, at present, known only from Canada. Possible other host plants in the genus *Heuchera* (Saxifragiaceae) are widespread in North America and the weevil may be more widely distributed than currently known. Whether or not a Canadian endemic, it is a little-known and possibly at-risk species that is worth searching for.

## Supplementary Material

XML Treatment for Euparius paganus

XML Treatment for Allandrus populi

XML Treatment for Trigonorhinus alternatus

XML Treatment for Trigonorhinus tomentosus tomentosus

XML Treatment for Gonotropis dorsalis

XML Treatment for Eusphyrus walshii

XML Treatment for Euxenus punctatus

XML Treatment for Loborhynchapion cyanitinctum

XML Treatment for Ischnopterapion (Ischnopterapion) loti

XML Treatment for Stenopterapion meliloti

XML Treatment for Trichapion nigrum

XML Treatment for Trichapion porcatum

XML Treatment for Nanophyes marmoratus marmoratus

XML Treatment for Sphenophorus incongruus

XML Treatment for Lissorhoptrus oryzophilus

XML Treatment for Anthonomus rufipes

XML Treatment for Anthonomus suturalis

XML Treatment for Anthonomus tectus

XML Treatment for Pseudanthonomus seriesetosus

XML Treatment for Cionus scrophulariae

XML Treatment for Curculio pardalis

XML Treatment for Curculio sulcatulus

XML Treatment for Mecinus janthinus

XML Treatment for Rhinusa neta

XML Treatment for Orchestes alni

XML Treatment for Promecotarsus densus

XML Treatment for Smicronyx griseus

XML Treatment for Smicronyx lineolatus

XML Treatment for Lignyodes bischoffi

XML Treatment for Lignyodes horridulus

XML Treatment for Tychius liljebladi

XML Treatment for Pnigodes setosus

XML Treatment for Barinus cribricollis

XML Treatment for Sibariops confinis

XML Treatment for Sibariops confusus

XML Treatment for Cosmobaris scolopacea

XML Treatment for Trichobaris trinotata

XML Treatment for Ampeloglypter ampelopsis

XML Treatment for Desmoglyptus crenatus

XML Treatment for Ceutorhynchus hamiltoni

XML Treatment for Microplontus campestris

XML Treatment for Dietzella zimmermanni

XML Treatment for Parenthis vestitus

XML Treatment for Pelenomus squamosus

XML Treatment for Asperosoma echinatum

XML Treatment for Psomus armatus

XML Treatment for Cylindrocopturus furnissi

XML Treatment for Cylindrocopturus quercus

XML Treatment for Caulophilus dubius

XML Treatment for Pseudopentarthrum parvicollis

XML Treatment for Rhyncolus knowltoni

XML Treatment for Rhyncolus macrops

XML Treatment for Acalles carinatus

XML Treatment for Cryptorhynchus tristis

XML Treatment for Tyloderma foveolatum

XML Treatment for Listronotus maculicollis

XML Treatment for Listronotus punctiger

XML Treatment for Cyrtepistomus castaneus

XML Treatment for Atrichonotus taeniatulus

XML Treatment for Otiorhynchus ligustici

XML Treatment for Larinus planus

XML Treatment for Magdalis inconspicua

XML Treatment for Magdalis salicis

XML Treatment for Microhyus setiger

XML Treatment for Dryocoetes autographus

XML Treatment for Dryocoetes caryi

XML Treatment for Lymantor alaskanus

XML Treatment for Hylastes macer

XML Treatment for Hylastes opacus

XML Treatment for Dendroctonus ponderosae

XML Treatment for Dendroctonus simplex

XML Treatment for Tomicus piniperda

XML Treatment for Ips perroti

XML Treatment for Hylocurus rudis

XML Treatment for Micracis suturalis

XML Treatment for Pseudothysanoes rigidus

XML Treatment for Phloeosinus pini

XML Treatment for Phloeotribus liminaris

XML Treatment for Phloetribus piceae

XML Treatment for Phloeotribus scabricollis

XML Treatment for Scolytus multistriatus

XML Treatment for Scolytus oregoni

XML Treatment for Scolytus piceae

XML Treatment for Scolytus rugulosus

XML Treatment for Scolytus schevyrewi

XML Treatment for Euwallacea validus

XML Treatment for Xyleborinus attenuatus

XML Treatment for Xyleborus affinis

XML Treatment for Xyleborus celsus

XML Treatment for Xyleborus ferrugineus

XML Treatment for Xylosandrus crassiusculus

XML Treatment for Xylosandrus germanus
